# Neonatal, infant, and child mortality among women exposed to intimate partner violence in East Africa: a multi-country analysis

**DOI:** 10.1186/s12905-019-0867-2

**Published:** 2020-01-23

**Authors:** Peter Memiah, Tristi Bond, Yvonne Opanga, Caroline Kingori, Courtney Cook, Michelle Mwangi, Nyawira Gitahi-Kamau, Deus Mubangizi, Kevin Owuor

**Affiliations:** 1 Division of Epidemiology and Prevention, Institute of Human Virology (IHV): University of Maryland School of Medicine, 725 W. Lombard Street, Baltimore, MD 21201 USA; 2 Amref Health Africa: Lang’ata Road, Amref Health Africa Headquarters, Nairobi, Kenya; 3 College of Health Sciences and Professions; Department of Social and Public Health, Ohio University Grover Center W347, 45701 Athens, OH USA; 4 BiologyDepartment, University of West Florida, Building 58, Room 79, 11000 University Parkway Pensacola, Pensacola, FL 32514 USA; 5 Department of Economics, University of Nairobi, P.O Box 30197-00100, GPO, Nairobi, Kenya; 6 Institute of Tropical Medicine and Infectious Disease, KEMRI, Kenya; 7 University of Maryland; School of Medicine, Baltimore, USA; 8 Centre for Health Solutions, Nairobi, Kenya

## Abstract

**Background:**

Most neonatal, infant, and child deaths occur in low- and middle-income countries (LMICs), where incidence of intimate partner violence (IPV) is highest in the world. Despite these facts, research regarding whether the two are associated is limited. The main objective was to examine associations between IPV amongst East African women and risk of death among their neonates, infants, and children, as well as related variables.

**Methods:**

Analysis was conducted on data drawn from the Demographic and Health Surveys (DHS) conducted by ICF Macro/MEASURE DHS in five East African countries: Burundi, Kenya, Rwanda, Tanzania, and Uganda. The analytical sample included 11,512 women of reproductive age (15–49 years). The outcome variables, described by proportions and frequencies, were the presence or absence of neonatal, infant, and under-five mortality. Our variable of interest, intimate partner violence, was a composite variable of physical, sexual, and emotional abuse; chi-square tests were used to analyze its relationship with categorical variables. Adjusted odds ratios (aOR) were also used in linking sexual autonomy to independent variables.

**Results:**

Children born to women who experienced IPV were significantly more likely to die as newborns (aOR = 1.3, 95% confidence interval [CI]: 1.4–2.2) and infants (aOR = 1.9, 95% CI: 1.6–2.2), and they were more likely to die by the age of five (aOR = 1.5, 95% CI: 1.01–1.55). Socioeconomic indicators including area of residence, wealth index, age of mother/husband, religion, level of education, employment status, and mass media usage were also significantly associated with IPV. After regression modelling, mothers who were currently using contraceptives were determined less likely to have their children die as newborns (aOR = 0.5, 95% CI: 0.3–0-7), as infants (aOR = 0.5, 95% CI: 0.3–06), and by age five (aOR = 0.4, 95% CI: 02–0.6).

**Conclusion:**

Understanding IPV as a risk indicator for neonatal, infant, and child deaths can help in determining appropriate interventions. IPV against women should be considered an urgent priority within programs and policies aimed at maximizing survival of infants and children in East Africa and the wellbeing and safety of their mothers.

## Background

According to the World Health Organization (WHO), 35% of women worldwide have experienced either sexual or physical violence at some point in their lifetime [1]. Intimate partner violence (IPV), which includes physical, sexual, and emotional abuse as well as controlling behaviors perpetrated by an intimate partner, is experienced by women globally within every culture, race, ethnicity, and throughout every socioeconomic class [2]. Incidents of violence against women are most commonly perpetrated by male intimate partners or ex-partners [2]. A WHO multi-country study on women’s health and domestic violence against women showed that among 24,000 women, a significant number of them experienced IPV – both physical and/or sexual - from their partners. Further, this study confirmed that IPV is a widespread, international issue as data was collected from women all over the world [3].

Individual, relationship, and community factors can increase the likelihood of a woman experiencing IPV [4–7]. Additionally, women may choose to stay in abusive relationships due to their economic situation, circumstances regarding their children, and other reasons that may not be conducive to leaving [8]. When women are subjected to IPV, unintended pregnancies, STIs, and other diseases and complications may occur [4]. Women subjected to IPV are not the only individuals negatively impacted from the violence. IPV can hinder the health and wellbeing of children and infants within the family [5, 6].

Most neonatal, infant, and child deaths occur in low- and middle-income countries (LMICs), where incidence of IPV is highest in the world [7]. Research on neonatal, infant, and child mortality and its respective causes are pertinent to decreasing mortality rates across the globe. In 2015, 4.5 million infants died within their first year of life, with sub-Saharan Africa having the highest infant mortality rate of 55 per 1000 live births [8].

The United Nation’s Sustainable Development Platform aims to collectively bring prosperity to all countries partnering for progress by setting 17 goals [9]. Sustainable Development Goal 5 aims to achieve gender equality while empowering all women and girls. By 2030, the hope is to eliminate all forms of discrimination and violence towards women as well as harmful practices of gender mutilation and forced marriages. Additionally, there should be equal opportunities for all women, access to quality sexual and reproductive healthcare, and adoption of sound government policies that will protect these rights [10]. Since 1999, The United Nations has successfully reduced infant mortality by more than 50%; yet IPV and neonatal, infant, and child mortality remain an active issue in the field of public health [9].

Burundi, Kenya, Rwanda, Tanzania, and Uganda are not immune to these statistics (see Table 1), although the countries have made progress in decreasing the mortality rates of neonates, infants, and children over time [11, 12]. Known factors contributing to high mortality within sub-Saharan Africa include: high population areas that put a strain on services, birth asphyxia, infections, AIDS, pneumonia, genocide, and diarrheal diseases [11, 12]. The health and well-being of the mother is a key component in the health and well-being of her children [12, 13]. Due to the impact and role a mother has in the development of her children, it is clear how IPV negatively affects the health of infants and children as well as impacting mortality among this age group. Our study, therefore, aims to examine associations between IPV against women and death rates among their neonates, infants, and children in East Africa.

**Table 1:** Country statistics

Country	Year	Population (no.)	GDP per Capita (USD)	Crude Birth Rate per 1000	Maternal Mortality per 100,000 Live Births	Under-5 Mortality per 1000 Live Births	Neonatal Mortality per 1000 Live Births	Sexual Violence 15–49 Years
Burundi	2015–2016	10.8 M	285.7	34	334	71.7	24.2	23%
Kenya	2014–2015	43 M	1143.1	30.5	362	52	22	14%
Rwanda	2015–2016	10.5 M	719	32.6	210	50	20	22.4%
Tanzania	2015–2016	50.1 M	867	37.2	398	67	25	17%
Uganda	2015–2016	41 M	580.4	42.1	432	64	27	39%

## Methods

This analysis was conducted using data from the Demographic and Health Survey (DHS), a series of nationally representative household studies being conducted in more than 90 countries by ICF Macro/MEASURE DHS with funding from the United States Agency for International Development [14]. DHS surveys collect household and individual data using standardized questionnaires and modules. This analysis uses DHS data collected from women of reproductive age (15–49 years) in Burundi (data collected in 2016–2017), Kenya (2014), Rwanda (2014–2015), Tanzania (2017), and Uganda (2016). Our data were drawn from the domestic violence module, which is administered to a subsample of DHS survey respondents. All countries included in our analysis had included the questions from the domestic violence module.

### Sample size and sampling techniques

The DHS survey in the five East African countries was conducted with nationally representative samples of households in each (Kenya, 36,430; Tanzania, 12,563; Uganda, 19,588; Rwanda, 12,699; and Burundi, 8596). The sample for this analysis, based on the response to our IPV outcome variable, was 11,512 women of reproductive age (15–49 years) as follows: Kenya 2432, Tanzania 2001, Uganda 3579, Rwanda 1479, and Burundi 2021.

### Conceptual framework and study variables

**Fig. 1:**
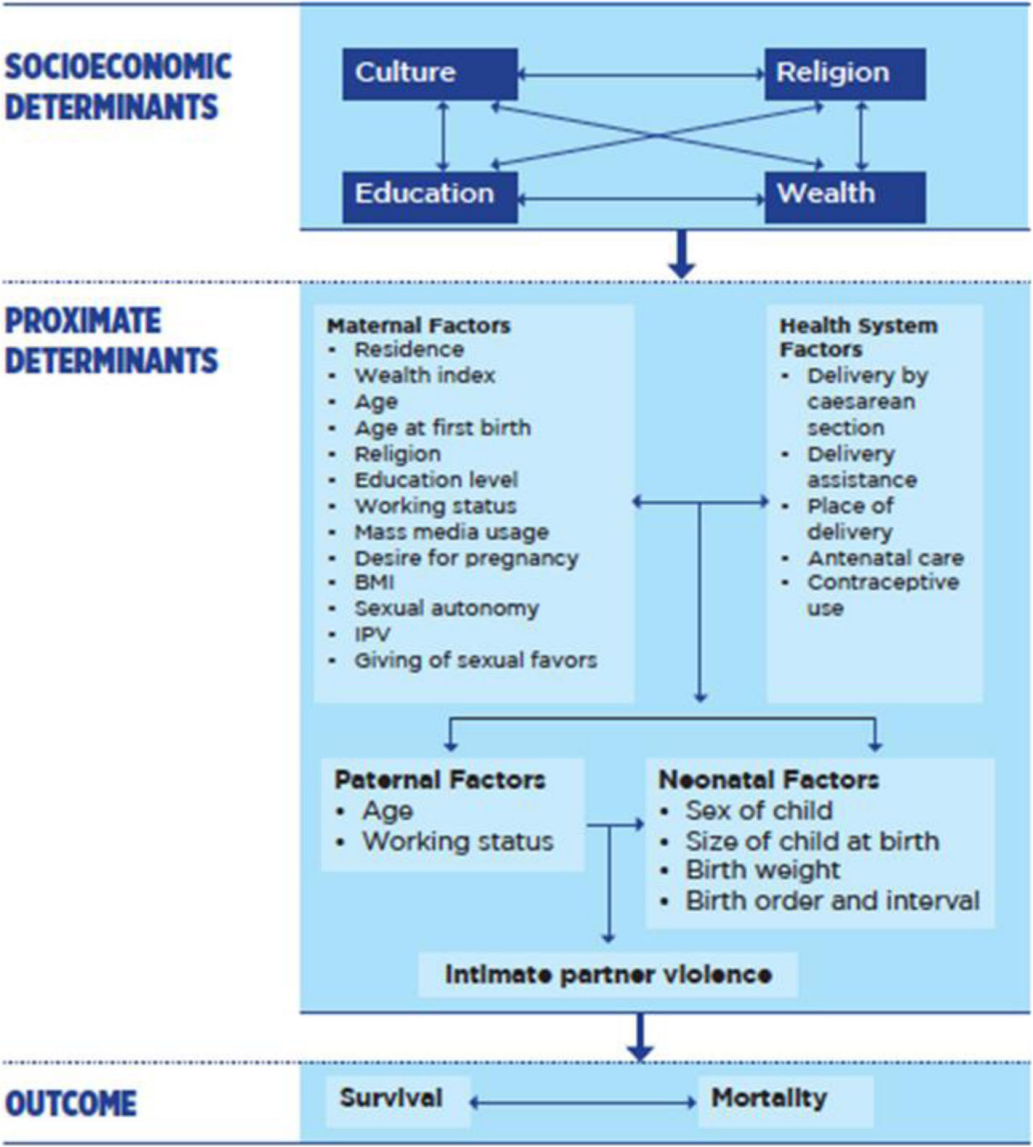
Conceptual Framework on Intimate Partner Violence

The study is organized by a conceptual framework which presents sociological and biological variables hierarchically into distal and proximate determinants of child survival in developing countries [15]. A literature search conducted using Google Scholar, PubMed, and Hinari was used to identify variables addressing the relationship between IPV and neonatal, infant, and child mortality in sub-Saharan Africa. Distal variables include a variety of socioeconomic determinants and community factors. Proximate factors include health status factors of both mother and neonate, infant, or child, including levels of IPV that contribute to infant, child, and neonatal mortality, as indicated in Fig. 1 and Table 2.

**Table 2:** Variable description and categorization

Variables	Description and Categorization
Socioeconomic Determinants	
Residence	Area of residence (1 = Rural; 2 = Urban)
Wealth index	Household wealth index (1 = Poor; 2 = Middle; 3 = Rich)
Mother’s age	Age of mother (1 = < 20; 2 = 20–29; 3 = 30–49)
Mother’s age at first birth	Age of mother at first birth (1 = < 20; 2 = 20–29, 3 = 30–49)
Mother’s religion	Mother’s religion (1 = Roman Catholic; 2 = Protestant/Other Christian; 3 = Muslim; 4 = No religion)
Mother’s education level	Mother’s education level (1 = No education; 2 = Primary; 3 = Secondary; 4 = Higher)
Mother’s working status	Mother’s working status (1 = No; 2 = Yes)
Mother’s mass media usage	Mother’s mass media usage (1 = Yes; 2 = No)
Maternal Factors	
Desire for pregnancy	Mother’s desire to have a baby (1 = Then; 2 = Later; 3 = No more)
BMI (kg/m^2^)	Mother’s body mass index (1 = < 18.5; 2= > =18.5):
Sexual autonomy	Mother’s consent to engage in sexual activities (1 = No; 2 = Yes)
Mother experienced IPV	Mother experienced IPV (1 = No; 2 = Yes)
Mother giving sexual favors	Mother seeking to provide sexual favors for economic gain (1 = No; 2 = Yes)
Health System Factors	
Delivery by Caesarean section	Delivery by Caesarean section (1 = No; 2 = Yes)
Delivery assistance	Professional assistance during birth (1 = Non-health professional; 2 = Health professional)
Place of delivery	Place of delivery (1 = Home delivery; 2 = Hospital/Other)
Antenatal care	Antenatal care received by mother during pregnancy (1 = No; 2 = Yes)
Contraceptive use at last sexual encounter	Mother’s contraceptive use (1 = No; 2 = Yes)
Paternal Factors	
Husband/partner’s age	Age of husband (1 = < 29; 2 = 30–39; 3 = 40–49; 4 = 50 plus)
Father’s working status	Father’s working status (1 = No; 2 = Yes)
Neonatal Factors	
Sex of child	Sex of child (1 = Male; 2 = Female)
Size of child at birth	Size of child at birth (1 = Average or larger; 2 = Small or very small; 3 = Missing)
Birth weight (g)	Weight of child at birth (1 = < 2500; 2 = 2500–3500; 3= > 3500; 4 = Not weighed; 5 = Don’t know)
Birth order and interval	Birth order and birth interval (1 = 2nd/3rd child, > 2 years; 2 = 1st child; 3 = 2nd/3rd child, <=2 years; 4 = 4th/higher child, > 2 years; 5 = 4th/higher child, <=2 years)

Neonatal mortality (NM) is the death of a neonate between birth and one month of life. Infant mortality (IM) is the death of an infant before his/her first birthday. Child mortality (under-five mortality/UM) is the death of infants and children under the age of five. These three types of mortality serve as the outcome variables in this study and were binary in nature. Neonatal death will be regarded as present (1 = if death occurs in the specified age period) or absent (0 = if the newborn/infant/child is alive in the specified age period).

The DHS instrument includes questions that ask ever-married women whether their current or most recent (if divorced, separated, or widowed) partner has ever perpetuated a series of behavioral items. The variable of interest was IPV, classified as a composite variable consisting of emotional, physical, and sexual violence. The composite variable took a binary form such that answering “Yes” to any of the forms of violence was regarded as present (1 = if respondent answered “Yes” to experiencing any of the forms of violence) or absent (0 = if respondent answered “No” to experiencing any of the forms of violence). Physical violence was described to respondents as when their (last) partner decided to: Push you, shake you, throw something at you, slap you, punch you, kick you, drag you, “beat you up”, try to choke or strangle you, burn you on purpose, threaten you with a knife or any other weapon, or attack you with a knife or any other weapon. Emotional violence was explained to respondents in the following way: Does/did he ever say or do something to humiliate you in front of others? Does he threaten you or someone close to you with harm? Does he become jealous or angry if you talk/talked to other men? Sexual violence was described to respondents as your partner ever having: Forced you to have sexual intercourse when you did not want to, forced you to perform any sexual acts you did not want to, or forced you to have sex with another person [16].

#### Other variables

DHS variables of age, marital status, educational level, religion, type and place of residence, occupational status, and wealth index were also used in our analysis. Additional sexual and behavioral variables for women were used and are indicated in Table 2.

Pre-calculated sampling weights which account for both sampling probability and non-response included in the datasets were applied. We also used the complex survey (svy) commands available within STATA 14 to account for clustered sampling design and to estimate robust standard errors as the basis for the 95% confidence intervals. Analysis involved descriptive statistics, specifically frequencies and percentages for all hypothesized correlates of IPV, and inferential statistics using Chi-square tests to assess bivariate association among IPV and covariates. Logistic regression analyses were used to assess for associations of the covariates to neonatal, infant, and child mortality reporting the odds ratios (OR) and respective 95% confidence intervals. Statistical levels of significance were evaluated at 5% as reported in the following sections.

## Results

Table 3 presents the estimated mortality rates (per 100) for neonates (NMR), infants (IMR), and children under five (UMR) among the five combined countries and further stratified by the characteristics of the sampled women listed in Table 2. The rates are presented with 95% probability confidence intervals. Most estimated rates for categories of a given demographic variable fall within the confidence intervals of the other categories, indicating no significant difference. This section highlights significant differences between categories of demographic variables.

**Table 3:** Neonatal, infant, and under-five mortality rates in East Africa (per 100 births) by demographic characteristics of mothers

Variable	East Africa (Kenya *n* = 2432, Uganda *n* = 3579, Tanzania *n* = 2001, Rwanda *n* = 1479, Burundi *n* = 2021, Total *n* = 11,512)		
	NMR (95% CI)	IMR (95% CI)	UMR (95% CI)
Intimate Partner Violence			
Mother Experienced IPV			
No	2.4 (2.2–2.7)	3.7 (3.4–4) 4.6 (4.3–5)	
Yes	2.9 (2.6–3.1)	4.2 (3.9–4.5)* 5.4 (5.1–5.7)*	
Socioeconomic Factors			
Residential area			
Rural	3 (2.6–3.4)	4.2 (3.7–4.7)*	5 (4.5–5.6)**
Urban	2.5 (2.3–2.6)	3.8 (3.6–4)	4.9 (4.7–5.1)
Wealth index			
Poor	2.5 (2.3–2.7)	4 (3.7–4.3)	5.3 (5–5.6)
Middle	2.5 (2.2–2.8)	3.7 (3.3–4.1)	4.6 (4.2–5)
Rich	2.8 (2.5–3.1)	3.9 (3.6–4.3)	4.7 (4.3–5.1)
Mother’s age			
< 20 years	3.8 (3.1–4.7)	4.9 (4.1–5.9)	5.6 (4.7–6.6)
20–29 years	2.5 (2.3–2.7)	3.8 (3.6–4.1)	4.9 (4.6–5.2)*
30–49 years	2.8 (2.5–3.1)	3.9 (3.7–4.2)	4.9 (4.6–5.3)
Mother’s age at first birth			
< 20 years	2.5 (2.3–2.7)	3.9 (3.7–4.2)	5.1 (4.8–5.4)
20–29 years	2.6 (2.4–2.9)*	3.9 (3.6–4.2)	4.7 (4.4–5)
30–49 years	3.2 (1.8–5.5)	4.7 (3.1–7.1)	5.5 (3.8–7.9)
Mother’s religion			
Roman Catholic	2.7 (2.4–3)	4.2 (3.8–4.5)	5.2 (4.8–5.7)
Protestant	2.4 (2.2–2.7)	3.7 (3.4–4)	4.6 (4.3–4.9)
Muslim	2.9 (2.4–3.6)	4 (3.4–4.8)	4.9 (4.2–5.7)
No religion	2.4 (1.9–3)	3.8 (3.2–4.5)	5.1 (4.4–5.9)
Mother’s education level			
No education	2.5 (2.2–2.8)	4.1 (3.8–4.6)	5.7 (5.2–6.2)
Primary	2.6 (2.5–2.8)	4 (3.8–4.3)	5 (5.8–5.3)
Secondary	2.5 (2.1–2.9)	3.5 (3–4)	4 (3.5–4.6)
Higher	2.8 (1.8–4.5)	3.5 (2.4–5.2)	3.7 (2.5–5.3)
Mother’s working status			
No	2.6 (2.3–3.1)	3.8 (3.4–4.3)	4.8 (4.3–5.3)
Yes	2.6 (2.5–2.8)	4 (3.8–4.3)	5.1 (4.8–5.4)
Mother’s mass media usage			
Yes	2.5 (2.2–2.8)	3.9 (3.6–4.3)	5.2 (4.8–5.6)
No	2.6 (2.5–2.8)	3.9 (3.7–4.2)	4.8 (4.6–5.1)
Maternal Factors			
Desire for pregnancy			
Then	2.8 (2.5–3)	4.2 (3.9–4.5)	5.3 (5–5.6)
Later	2.3 (2–2.6)*	3.5 (3.1–3.8)	4.4 (4–4.9)*
No more	2.7 (2.2–3.4)	3.8 (3.2–4.6)	4.5 (3.8–5.3)
Mother’s BMI			
< 18.5	1.7 (1.3–2.3)	3.1 (2.4–3.9)	4.1 (3.4–5)
> =18.5	2.8 (2.6–3.1)	4.1 (3.9–4.4)*	5.2 (4.9–5.5)*
Sexual autonomy			
No	3.1 (2.7–3.6)	4.6 (4.1–5.1)	5.7 (5.1–6.2)
Yes	2.5 (2.3–2.7)	3.8 (3.5–4)	4.8 (4.5–5)
Mother giving sexual favors			
No	2.6 (2.1–3.2)	3.9 (3.3–4.6)	4.6 (4–5.3)
Yes	3.2 (1.3–7.5)	5.3 (2.1–12.9)	5.6 (2.3–13)
Health System Factors			
Delivery by Caesarean section			
No	2.5 (2.4–2.7)	3.8 (3.7–4.1)	4.9 (4.7–5.1)
Yes	3.7 (2.9–4.7)*	4.8 (3.9–5.9)*	5.7 (4.8–6.8)*
Delivery assistance			
Non-health professional	2.7 (2.4–3)	4 (3.7–4.4)	5.1 (4.7–5.6)
Health professional	2.5 (2.3–2.7)	3.7 (3.5–4)	4.7 (4.4–5)
Place of delivery			
Home delivery	2.6 (2.3–2.9)	4 (3.7–4.4)	5.2 (4.8–5.6)
Hospital/other	2.6 (2.4–2.8)	3.8 (3.6–4.1)	4.8 (4.5–5)
Antenatal care			
No	4.9 (3.6–6.6)	6.6 (5.1–8.5)	7.4 (5.8–9.3)
Yes	1.7 (1.5–1.8)	2.6 (2.4–2.7)	3.1 (2.9–3.3)
Contraceptive use			
Not using	3 (2.8–3.2)	4.6 (4.3–4.8)	5.8 (5.6–6.2)
Using	2.1 (1.8–2.3)*	3 (2.8–3.3)*	3.7 (3.4–4)*
Paternal Factors			
Husband/partner’s age			
< 29 years	2.9 (2.5–3.3)	4.1 (3.7–4.6)	5.3 (4.8–5.8)
30–39 years	2.3 (2.1–2.6)	3.7 (3.4–4)	4.6 (4.2–5)
40–49 years	2.8 (2.4–3.2)	4 (3.6–4.5)	5 (4.5–5.5)
50 plus years	2.9 (2.3–3.7)	4.3 (3.5–5.2)	6 (5.1–7)
Father’s working status			
No	3.4 (2.3–5)	4.9 (3.6–6.7)	6.5 (5–8.3)
Yes	2.6 (2.4–2.8)	3.9 (3.7–4.1)	4.9 (4.7–5.2)
Neonatal Factors			
Sex of child			
Male	2.9 (2.7–3.1)	4.3 (4–4.6)	5.3 (5.1–5.7)
Female	2.3 (2.1–2.5)	3.5 (3.3–3.8)	4.5 (4.2–4.8)
Size of child at birth			
Average or larger	2.1 (1.9–2.3)	3.4 (3.2–3.6)	4.4 (4.2–4.6)
Small or very small	4.8 (4.2–5.3)*	6.4 (5.8–7.1)*	7.6 (7–8.3)*
Don’t know	12.2 (9.3–15.9)*	13.1 (10.1–16.8)*	14.6 (11.6–18.2)*
Birth weight (g)			
< 2500	6.5 (5.5–7.5)	8.9 (7.7–10.1)	10.2 (9–11.6)
2500–3500	1.5 (1.4–1.7)*	2.7 (2.5–3)*	3.6 (3.4–3.9)*
> 3500	1.9 (1.6–2.3)*	3.1 (2.7–3.6)	4.2 (3.7–4.7)
Not weighed	3.8 (3.4–4.2)	5.1 (4.7–5.6)	6.3 (5.9–6.8)
Don’t know	16.3(12.8–20.5)*	17.8(14.2–22.1)	19.3(15.5–23.6)
Birth order and interval			
2nd/3rd child, > 2 years	2 (1.7–2.2)	3.2 (2.9–3.5)	4.1 (3.8–4.5)
1st child	3.3 (2.9–3.7)	4.4 (4–4.9)	5.4 (5–5.9)
2nd/3rd child, ≤2 years	2.6 (2.1–3.1)	4 (3.4–4.6)	5.2 (4.6–5.9)
4th/higher child, > 2 years	2.3 (2.1–2.6)	3.6 (3.2–3.9)	4.5 (4.2–4.9)
4th/higher child, ≤2 years	3.7 (3.2–4.3)*	6 (5.3–6.8)*	7.3 (6.5–8.1)*

Two asterisks (**) denotes the *p*-value is <0.001, and one asterisk (*) denotes the *p*-value is <0.05

Our results indicate that the birth weight of neonates, infants, and children under five was a significant factor in child mortality – especially considering that small or very small babies had higher mortality rates (NMR: 4.8 vs. 2.1; IMR: 6.4 vs. 3.4; UMR: 7.6 vs. 4.4). Additionally, the mortality rates of offspring of women who did not receive antenatal care was higher than those who received antenatal care (NMR: 4.9 vs. 1.7; IMR: 6.6 vs. 2.6; UMR: 7.4 vs. 3.1). It should be noted that there was a small group of women in this category causing the confidence intervals to be larger. The rates of neonatal mortality were higher for babies delivered by Caesarean section (NMR: 3.7 vs. 2.5), and the estimated infant and under five rates for Caesarean babies were also higher but within confidence intervals of the non-Caesarean delivery estimates. Furthermore, women who reported IPV showed higher rates of child mortality – most notably under five mortality rates were significantly higher (5.4 vs. 4.6).

### Characteristics of women who experience IPV: bivariate comparisons

Tests for independence evidenced that socioeconomic indicators like area of residence, wealth index, age of mother/husband, religion, level of education, employment status, and mass media usage were significantly associated with IPV. Women who had a body mass index (BMI) lower than 18.5 kg/m^2^ and desired pregnancy later than at the time of interview were associated with a lower proportion of experiencing IPV (*p* < 0.001). Although the association between sex of the child and IPV was not significant, perceived and actual birth weight were significantly associated with IPV. More so, IPV was significantly associated with child birth order, number, and interval (*p* < 0.001). However, there was no significant association between neonatal, infant, and under-five mortality between the outcome categories of IPV. Women who delivered in hospitals via Caesarean section received professional assistance during birth, and using contraceptives was associated with lower proportions of IPV (*p* < 0.001). IPV was also significantly associated with antenatal care and sexual autonomy (*p* < 0.001). Mothers receiving money/gifts/favors in exchange for sex were not significantly associated with IPV. These findings are presented in Table 4.

**Table 4:** Characteristics of women who experience intimate partner violence: bivariate comparisons

Variable	Intimate Partner Violence (IPV)		Pearson chi2 (*p* value)
	No, n (%) (*n* = 4780)	Yes, n (%) (*n* = 5390)	
Socioeconomic Determinants			
Residential area			
Urban	1864 (39)	1471 (27.7)	144.836 (< 0.001)*
Rural	2916 (61)	3838 (72.3)	
Wealth index			520.722 (< 0.001)*
Poor	2095 (43.8)	3404 (64.1)	
Middle	837 (17.5)	878 (16.5)	
Rich	1848 (38.7)	1027 (19.3)	
Mother’s age			15.468 (< 0.001)*
< 20 years	203 (4.2)	284 (5.3)	
20–29 years	2535 (53)	2934 (55.3)	
30–49 years	2042 (42.7)	2091 (39.4)	
Mother’s age at first birth			85.857 (< 0.001)*
< 20 years	2561 (53.6)	3318 (62.5)	
20–29 years	2153 (45)	1950 (36.7)	
30–49 years	66 (1.4)	41 (0.8)	
Mother’s religion			37.736 (< 0.001)*
Roman Catholic	917 (19.2)	970 (18.3)	
Protestant/Other Christian	3011 (63)	3201 (60.3)	
Muslim	747 (15.6)	916 (17.3)	
No religion	105 (2.2)	220 (4.1)	
Mother’s education level			451.636 (< 0.001)*
No education	790 (16.5)	1396 (26.3)	
Primary	2399 (50.2)	2964 (55.8)	
Secondary	1105 (23.1)	821 (15.5)	
Higher	486 (10.2)	128 (2.4)	
Mother’s working status			4.446 (0.035)*
No	1695 (35.6)	1992 (37.7)	
Yes	3061 (64.4)	3296 (62.3)	
Mother’s mass media usage			108.929 (< 0.001)*
No	978 (20.5)	1566 (29.5)	
Yes	3802 (79.5)	3743 (70.5)	
Maternal Factors			
Desire for pregnancy			16.520 (< 0.001)*
Then	3297 (69.2)	3468 (65.5)	
Later	1035 (21.7)	1268 (23.9)	
No more	430 (9)	559 (10.6)	
Mother’s BMI			46.377 (< 0.001)*
< 18.5	449 (9.5)	729 (13.9)	
> =18.5	4284 (90.5)	4519 (86.1)	
Sexual autonomy			28.238 (< 0.001)*
No	1043 (22)	1387 (26.6)	
Yes	3691 (78)	3824 (73.4)	
Mother giving sexual favors			0.506 (0.477)
No	4223 (97)	4625 (96.8)	
Yes	129 (3)	154 (3.2)	
Health System Factors			
Delivery by Caesarean section			64.548 (< 0.001)*
No	4360 (91.4)	5046 (95.3)	
Yes	412 (8.6)	247 (4.7)	
Delivery assistance			268.011 (< 0.001)*
Non-health professional	1547 (33.8)	2537 (50.4)	
Health professional	3024 (66.2)	2499 (49.6)	
Place of delivery			264.970 (< 0.001)*
Home delivery	1752 (36.8)	2804 (53)	
Hospital/other	3010 (63.2)	2489 (47)	
Antenatal care			54.979 (< 0.001)*
No	129 (3.7)	281 (7.7)	
Yes	3398 (96.3)	3355 (92.3)	
Contraceptive use			109.172 (< 0.001)*
Not using	2257 (47.2)	3059 (57.6)	
Using	2523 (52.8)	2250 (42.4)	
Paternal Factors			
Husband/partner’s age			109.172 (< 0.001)*
< 29 years	851 (21.5)	1111 (24.6)	
30–39 years	1892 (47.7)	1874 (41.5)	
40–49 years	883 (22.3)	990 (21.9)	
Father’s working status			50.598 (< 0.001)*
No	51 (1.2)	129 (2.6)	
Yes	4362 (98.8)	4783 (97.4)	
Neonatal Factors			
Sex of child			1.464 (0.226)
Male	2449 (51.2)	2656 (50)	
Female	2331 (48.8)	2653 (50)	
Size of child at birth			9.008 (0.011)*
Average or larger	3941 (82.7)	4326 (81.7)	
Small or very small	777 (16.3)	881 (16.6)	
Don’t know	47 (1)	88 (1.7)	
Birth weight (Kg)			325.327 (< 0.001)*
< 2500	237 (5)	208 (3.9)	
2500–3500	2201 (46.3)	1730 (32.7)	
> 3500	799 (16.8)	736 (13.9)	
Not weighed	1491 (31.3)	2545 (48.1)	
Don’t know	29 (0.6)	69 (1.3)	
Birth order and interval			86.056 (< 0.001)*
2nd/3rd child, > 2 years	1513 (31.7)	1472 (27.7)	
1st child	1201 (25.1)	1070 (20.2)	
2nd/3rd child, ≤2 years	322 (6.7)	490 (9.2)	
4th/higher child, > 2 years	1345 (28.1)	1751 (33)	
4th/higher child, ≤2 years	399 (8.3)	526 (9.9)	
Neonatal mortality			1.437 (0.231)
Alive	4675 (97.8)	5173 (97.4)	
Dead	105 (2.2)	136 (2.6)	
Infant mortality			0.099 (0.753)
Alive	4618 (96.6)	5123 (96.5)	
Dead	162 (3.4)	186 (3.5)	
Under-five mortality			0.933 (0.334)
Alive	4593 (96.1)	5081 (95.7)	
Dead	187 (3.9)	228 (4.3)	

One asterisk (*) denotes the *p*-value is <0.05

Women who are between ages 20 and 29, who reside in rural areas, who do not use mass media at all, who had a BMI of greater than or equal to 18.5 kg/m^2^, and who were not aware of the birthweight of their babies, were significantly more likely to experience infant and under-five mortality among their children. Additionally, children were more at risk of dying as infants and prior to the age of five if their mothers experienced IPV. However, antenatal care and contraceptive use decreased the likelihood of infant and children under five deaths. Results from both the unadjusted and adjusted regression analyses are presented in Table 5.

**Table 5:** Unadjusted and adjusted regression analysis

	Unadjusted and Adjusted Regression Analysis					
Variable	Unadjusted Analysis			Adjusted Analysis		
	Neonatal Mortality	Infant Mortality	Under-five mortality	Neonatal Mortality	Infant Mortality	Under-five mortality
Intimate Partner Violence						
Mother experienced IPV						
No	Ref	Ref	Ref	Ref	Ref	Ref
Yes	1.114 (0.744–1.668)	1.886 (1.639–1.930)*	1.976 (1.727–2.11)*	1.340 (1.408–2.222)*	1.999 (1.656–2.22)*	1.057 (1.01–1.550)*
Residential area						
Urban	Ref	Ref	Ref			Ref
Rural	1.860 (1.630–1.951)	1.807 (1.640–1.991)*	1.826 (1.71–1.916)**			1.704 (1.458–1.88)**
Wealth index						
Poor	Ref	Ref	Ref	Ref		
Middle	0.84 (0.811–1.531)	0.970 (0.729–1.292)	0.960 (0.749–1.230)	0.965 (0.515–1.806)		
Rich	0.806 (0.798–1.532)	0.830 (0.879–1.452)	0.94 (0.793–1.259)	0.493 (0.270–0.902)*		
Mother’s age						
< 20 years	Ref	Ref	Ref	Ref		
20–29 years	1.549 (0.738–3.251)	1.584 (0.889–2.822)	1.694 (1.002–2.862)*	0.799 (0.247–2.585)		
30–49 years	1.684 (0.806–3.516)	1.551 (0.884–2.721)	1.654 (0.991–2.763)	1.157 (0.359–3.735)		
Mother’s age at first birth						
< 20 years	Ref	Ref	Ref			
20–29 years	1.321 (1.001–1.745)*	1.092 (0.866–1.377)	1.002 (0.812–1.236)			
30–49 years	1.902 (0.495–7.305)	1.481 (0.482–4.551)	1.275 (0.445–3.656)			
Mother’s religion						
Roman Catholic	Ref	Ref	Ref			
Protestant/Other Christian	1.000 (0.711–1.408)	1.057 (0.790–1.413)	1.095 (0.831–1.444)			
Muslim	1.223 (0.783–1.910)	1.133 (0.752–1.709)	1.124 (0.765–1.650)			
No religion	1.189 (0.676–2.090)	0.962 (0.580–1.594)	1.031 (0.670–1.586)			
Mother’s education level						
No education	Ref	Ref	Ref			
Primary	1.016 (0.748–1.382)	1.159 (0.884–1.519)	1.212 (0.949–1.547)			
Secondary	0.826 (0.511–1.333)	1.117 (0.784–1.592)	1.063 (0.773–1.463)			
Higher	1.421 (0.722–2.796)	1.266 (0.702–2.283)	1.109 (0.634–1.939)			
Mother’s working status						
No	Ref	Ref	Ref			
Yes	1.006 (0.625–1.618)	1.022 (0.687–1.519)	1.051 (0.737–1.498)			
Mother’s mass media usage						
Yes	Ref	Ref	Ref	Ref	Ref	Ref
Not at All	1.312 (0.970–1.775)	1.405 (1.096–1.803)*	1.186 (0.936–1.502)	2.715 (1.392–5.292)*	1.697 (1.001–2.877)*	1.599 (0.942–2.715)
Maternal Factors						
Desire for pregnancy						
Then	Ref	Ref	Ref			
Later	0.558 (0.339–0.921)*	0.682 (0.443–1.050)	0.681 (0.465–0.999)*			
No more	1.124 (0.656–1.927)	1.012 (0.632–1.621)	0.954 (0.618–1.473)			
Mother’s BMI						
< 18.5	Ref	Ref	Ref	Ref	Ref	Ref
> =18.5	1.237 (0.734–2.086)	1.607 (1.015–2.545)*	1.572 (1.036–2.386)*	2.445 (1.071–5.583)*	2.668 (1.304–5.457)*	2.383 (1.206–4.710)*
Mother giving sexual favors						
No	Ref	Ref	Ref			
Yes	1.245 (0.496–3.126)	1.255 (0.429–3.669)	1.147 (0.417–3.156)			
Health System Factors						
Delivery by Caesarean section						
No	Ref	Ref	Ref	Ref		
Yes	1.551 (0.821–2.929)	1.351 (0.819–2.28)	1.332 (0.858–2.069)	2.108 (0.894–4.971)		
Delivery assistance						
Non-health professional	Ref	Ref	Ref			
Health professional	1.002 (0.740–1.357)	1.049 (0.823–1.337)	0.999 (0.805–1.240)			
Place of delivery						
Home delivery	Ref	Ref	Ref			
Hospital/other	0.996 (0.748–1.326)	1.030 (0.818–1.296)	0.967 (0.787–1.189)			
Antenatal care						
No	Ref	Ref	Ref	Ref	Ref	Ref
Yes	0.258 (0.157–0.425)*	0.347 (0.229–0.525)*	0.368 (0.246–0.549)*	0.418 (0.200–0.875)*	0.528 (0.269–1.035)	0.614 (0.315–1.196)
Contraceptive use						
Not using	Ref	Ref	Ref	Ref	Ref	Ref
Using	0.683 (0.513–0.910)*	0.671 (0.534–0.841)*	0.622 (0.507–0.763)*	0.409 (0.248–0.673)*	0.404 (0.254–0.644)*	0.370 (0.233–0.589)*
Paternal Factors						
Husband/partner’s age						
< 29 years	Ref	Ref	Ref			
30–39 years	0.626 (0.342–1.146)	0.716 (0.438–1.170)	0.726 (0.467–1.127)			
40–49 years	0.758 (0.406–1.413)	0.761 (0.459–1.259)	0.734 (0.461–1.166)			
50 plus years	0.950 (0.449–2.007)	0.743 (0.387–1.427)	0.829 (0.469–1.464)			
Father’s working status						
No	Ref	Ref	Ref			
Yes	1.248 (0.402–3.874)	1.645 (0.604–4.484)	1.486 (0.596–3.700)			
Sex of child						
Male	Ref	Ref	Ref			
Female	0.837 (0.647–1.084)	0.852 (0.689–1.052)	0.897 (0.741–1.086)			
Size of child at birth						
Average or larger	Ref	Ref	Ref	Ref	Ref	Ref
Small or very small	2.328 (1.555–3.487)*	1.703 (1.209–2.399)*	1.739 (1.263–2.394)*	2.202 (1.350–3.592)*	1.659 (1.047–2.630)*	1.564 (1.003–2.438)*
Don’t know	4.925 (1.852–13.09)*	2.897 (1.110–7.560)*	2.610 (1.044–6.526)*	5.792 (1.730–19.39)*	4.288 (1.108–16.60)*	3.440 (0.883–13.39)
Birth weight (Kg)						
< 2500	Ref	Ref	Ref			
2500–3500	0.226 (0.126–0.408)*	0.276 (0.145–0.526)*	0.273 (0.151–0.494)*			
> 3500	0.382 (0.147–0.994)*	0.556 (0.254–1.218)	0.530 (0.259–1.087)			
Not weighed	0.755 (0.402–1.417)	0.657 (0.343–1.259)	0.648 (0.360–1.167)			
Don’t know	3.781 (1.219–11.73)*	2.846 (0.954–8.492)	2.363 (0.816–6.841)			
Birth order and interval						
2nd/3rd child, > 2 years	Ref	Ref	Ref			Ref
1st child	1.285 (0.937–1.761)	1.128 (0.842–1.513)	1.112 (0.848–1.459)			0.608 (0.309–1.195)
2nd/3rd child, ≤2 years	0.990 (0.641–1.529)	0.966 (0.675–1.383)	0.932 (0.666–1.305)			0.615 (0.250–1.513)
4th/higher child, > 2 years	1.028 (0.749–1.411)	0.941 (0.723–1.225)	0.978 (0.768–1.245)			1.075 (0.675–1.713)
4th/higher child, ≤2 years	1.713 (1.203–2.439)*	1.824 (1.313–2.533)*	1.869 (1.377–2.537)*			1.945 (0.874–4.330)

Two asterisks (**) denotes the *p*-value is <0.001, and one asterisk (*) denotes the *p*-value is <0.05

## Discussion

The current study examined the association between IPV among women and rates of mortality among their neonates, infants, and children less than five years of age in East Africa using DHS, a nationally representative dataset. In resource-limited countries, such as Kenya, Burundi, Tanzania, Uganda and Rwanda, high mortality rates before a child reaches the age of five years are common [12]. Based on the adjusted model, our study findings found that women exposed to IPV were more likely to lose their children. Study findings provide evidence for an association showing that children born to women who are IPV survivors were significantly more likely to die as newborns, infants, and children under five. These findings are consistent with those of previous studies in Bangladesh [16], India [17], and Kenya, Egypt, Malawi, Honduras, & Rwanda [18]. In essence, we concur that the high infant and child mortality rates among women experiencing IPV could be influenced by the negative impact on their mental and physical health, which is likely to affect their pregnancy and subsequent caregiving [19].

There is evidence on the negative effects of IPV during pregnancy. A multi-country study by WHO involving 10 countries indicated a prevalence of 4–12% in most countries with 28% reported in Peru [2]. Another study involving 19 countries reported a higher prevalence of IPV during pregnancy in African and Latin American countries with highest prevalence of 13.5% with most victims being in the younger age groups (15–35 years) [19]. Studies indicate that IPV during pregnancy can result in antepartum hospitalization, pregnancy complications, low birth weight, and even infant death [20]. Further studies indicate that IPV during pregnancy is associated with miscarriage, late entry into prenatal care, stillbirth, premature labor, and fetal injury [2]. These findings support the negative impact of IPV on the health of the mother, which effects far more than just physical health and wellbeing. IPV affects the social, mental, and psychological well-being of the mother due to the traumatic nature of the violence they experience. This, therefore, affects their capacity to care for their child and contributes to underutilization of maternal health services [16]. Given the role that mothers play in a child’s life, their health status during and after pregnancy is a direct predictor of an infant and child’s health outcomes.

Violence against women can be linked to mortality rates among infants and children via various mechanisms. For instance, while socioeconomic indicators like area of residence, wealth index, age of mother/husband, religion, level of education, employment status, and mass media usage were significantly associated with IPV in the study, they can also be considered underlying factors in infant and child mortality. From these findings, the influence of social determinants of health in the relationship between IPV and child mortality is cause for further investigation. Interventions should strive to focus on addressing IPV at multiple levels and not just individual levels. The interconnectedness of the individual and environmental influences warrants an in-depth assessment of this association.

From the results of our study, various socioeconomic and demographic confounding factors revealed a significant influence in the association between IPV and mortality rates among neonates, infants, and children under five, including BMI and contraceptive use. Current findings revealed that women reporting a low BMI and chose to delay pregnancy were less likely to experience IPV. Furthermore, mortality rates in neonates, infants, and children under five were more likely to be reported among women who had a high BMI (above 18.5 kg/m^2^) and among those not using contraceptives. It has been shown that women who experienced IPV were more likely to raise children who were underweight and might experience stunted growth; additionally, there is a link between maternal BMI and wasting syndrome in children [21]. Underweight and wasting in children are risk factors for infant and child mortality [22]. In our study, perceived and actual birth weights were significantly associated with IPV. With regard to the association between contraceptive use and IPV, documented research provides conflicting evidence. Consistent with our findings, documented evidence supports that women who suffer from IPV were less likely to use contraceptives compared to women using contraceptives with less likelihood of finding themselves victims of IPV [23, 24]. We speculate that women who suffer from IPV are likely to use contraceptives because they do not want to raise children under such conditions.

Based on the findings of this study and prior documented research, IPV should be considered an important factor associated with neonatal, infant, and child mortality. It is paramount to provide adequate care to mothers during and after pregnancy to ensure the survival of children. Without such care, IPV can cause mothers to be unsuccessful in carrying a pregnancy to full term or in providing adequate care for their children. Adequate care can be provided by ensuring that mothers have access to maternal health care services and are trained to negotiate for contraceptive use and safer sex in order to enhance sexual autonomy [25, 26]. WHO has provided guidance on the role of the health system in managing and combatting IPV. Health care providers’ responses to those who have been victims of intimate partner violence must be multifaceted with identifying those at risk as only the first step in proper response. As providers screen patients, victims of IPV can explore their options regarding plans for safety and access to support services [27]. Study findings revealed that while IPV was significantly associated with death among neonates, infants, and children, other factors such as antenatal care, sexual autonomy, women delivering in hospitals, and receiving professional assistance during birth were protective factors. Consequently, it can be argued that access to adequate maternal health services can help bridge the gap between IPV and infant and child mortality rates.

## Limitations

The study relied on DHS data that is retrospective in nature. There is a risk of reporting and recall bias due to the reliance on the memory of an event that occurred in the past. Given the stigma surrounding IPV in sub-Saharan Africa, IPV exposure is underreported, which limits the generalizability of these findings across the countries analyzed. In addition, the cross-sectional nature of DHS data limits the likelihood of showing causal relations between IPV against women and neonatal, infant, and children under-five mortality rates. Also, it is challenging to determine whether the mortality rates preceded IPV or vice versa. Despite the limitations, this study examines different multi-level variables in relation to IPV. These findings provide a snapshot of the current association between IPV and infant and child mortality rates and can be used to facilitate contextualized interventions and strategies across East African countries.

## Conclusion

Our study findings revealed that women exposed to IPV were more likely to experience the death of a child. Antenatal care, sexual autonomy, perceived and actual birth weight, child birth order/interval, and socioeconomic indicators were significantly associated with IPV. This study revealed that IPV plays a negative role in neonatal, infant, and child mortality in East Africa and other resource-limited countries. To that end, IPV against women is a public health issue that not only negatively affects mothers but their children as well. Current and future interventions can only be sustained by adequate funding and policies that support women’s and children’s rights.

## Data Availability

Data was requested from the DHS program via email (https://dhsprogram.com/what-we-do/survey-Types/dHs.cfm). The data used for this study can be obtained through DHS (http://dhsprogram.com/) and are available upon request from the corresponding author.

## Abbreviations

AIDS: Acquired immune deficiency syndrome
BMI: Body mass index
CI: Confidence interval
DHS: Demographic Health Survey
HIV: Human immunodeficiency virus
IM: Infant mortality
IMR: Infant mortality rate
IPV: Intimate partner violence
LMIC: Low- and middle-income country
NM: Neonatal mortality
NMR: Neonatal mortality rate
OR (aOR): (Adjusted) odds ratio
UM: Under-five mortality
UMR: Under-five mortality rate
WHO: World Health Organization
